# GATA3 Expression Is a Poor Prognostic Factor in Soft Tissue Sarcomas

**DOI:** 10.1371/journal.pone.0156524

**Published:** 2016-06-01

**Authors:** Toshiaki Haraguchi, Hiroaki Miyoshi, Koji Hiraoka, Shintaro Yokoyama, Yukinao Ishibashi, Toshihiro Hashiguchi, Koutaro Matsuda, Tetsuya Hamada, Takahiro Okawa, Naoto Shiba, Koichi Ohshima

**Affiliations:** 1 Department of Pathology, Kurume University School of Medicine, Kurume, Fukuoka, Japan; 2 Department of Orthopedic Surgery, Kurume University School of Medicine, Kurume, Fukuoka, Japan; 3 Department of Surgery, Kurume University School of Medicine, Kurume, Fukuoka, Japan; Yokohama City University School of Medicine, JAPAN

## Abstract

**Objective:**

Recent studies have investigated the significance of GATA3 expression in patients with various malignant tumors. However, no previous studies have evaluated the clinicopathological importance of GATA3 expression in soft tissue sarcomas (STS) patients.

**Methods:**

We evaluated GATA3 expression in 76 STS cases using immunohistochemical analysis, and statistically compared clinicopathological characteristics between GATA3-positive and GATA3-negative cases.

**Result:**

GATA3-positive expression was significantly associated with a higher mitotic count (*P* < 0.0001). Disease-free survival (DFS) of GATA3-positive cases was significantly shorter than that of cases without GATA3 expression (*P* = 0.0104). Overall survival (OS) of GATA3-positive cases was significantly shorter than that of cases without GATA3 expression (*P* = 0.0006). GATA3-positive expression was significantly associated with shorter DFS in both univariate analysis (hazard ratio [HR], 2.719; *P* = 0.012) and multivariate analysis (HR, 2.711; *P* = 0.014). GATA3-positive expression was also significantly associated with worse OS in both univariate analysis (HR, 5.730; P = 0.0007) and multivariate analysis (HR, 5.789; P = 0.0008).

**Conclusion:**

These results indicate that GATA3 is an independent prognostic factor and suggest that evaluation of GATA3 expression might enable more effective clinical follow-up using prognostic stratification of STS patients.

## Introduction

Soft tissue sarcomas (STS), which involve mesenchymal cells, are malignant tumors that occur throughout the body. It accounts for < 1% of all malignant tumors; nevertheless, it frequently invades surrounding tissue and metastasizes to distant organs.[1,2] Surgical resection is recommended for localized STS, although approximately half of patients experience recurrence even though complete resection has been performed.[3] Moreover, one-third of patients eventually die from their STS tumors.

STS tumors are graded according to the French Fédération Nationale des Centres de Lutte Contre le Cancer (FNCLCC) system, in which grading is based on all sarcomas being considered as a single entity, because STS is rare and has many histological types.[4] However, this grading system does not work well for all types of sarcomas.[5]

GATA3 is a transcription factor belonging to the GATA family, members of which bind to the consensus DNA sequence G-A-T-A via zinc finger domains.[6] GATA3 expression is not observed in normal mesenchymal tissue.[7] Previous studies have suggested the important role of GATA3 in the proliferation and differentiation phases in a variety of normal tissue and organs.[6] In T-cell development, GATA3 is well-known to be an essential transcription factor in the differentiation of naive T cells to Th2 cells.[8] Additional GATA3 functions, including maintaining differentiation, adhesion, and proliferation of epithelial cells in tissues such as the mammary gland and skin, have also been reported,[9–12] as well as a role in the development of sympathetic neurons.[13]

Recent studies have reported GATA3 expression in neoplastic cells in patients with various malignant tumors, including breast cancer, gastric cancer, and neuroblastoma.[14–17] Some studies reported that decreased GATA3 expression in neoplastic cells compared to non-neoplastic cells was associated with poorer overall survival (OS) in breast cancer and gastric cancer. In contrast, studies in neuroblastoma indicated that increased GATA3 expression may be a poor prognostic marker for OS.[18] On the other hand, GATA3 expression was reported to be associated with expression of cyclinD1, HER2, and FOXO1, which might cause a worse clinical outcome. [17,19–21]

Only a few reports of GATA3 expression in neoplastic cells have been published in patients with mesenchymal tumors.[7] A patient with biphasic synovial sarcoma showed sporadic GATA3 expression; in contrast, focal to extensive expression was observed in patients with myxofibrosarcoma, undifferentiated/unclassified sarcomas, poorly differentiated angiosarcoma, leiomyosarcoma, and malignant peripheral nerve sheath tumor. It is remarkable that no previous studies have discussed the clinicopathological and prognostic importance of GATA3 expression in neoplastic cells of STS.

In this study, we investigated GATA3 expression using immunohistochemical (IHC) analysis, and evaluated the statistical association between this expression and clinicopathological features in STS cases.

## Materials and Methods

### Patients and samples

We reviewed formalin-fixed paraffin-embedded (FFPE) tissue samples from 76 STS patients who underwent tumor resection at the Department of Orthopedic Surgery in Kurume University Hospital from July 1998 to August 2014. Most of the patients in this study were included in the authors’ previous study.[22] In all cases, the pathological diagnoses were reviewed by 2 pathologists (OK and MH), according to the 2013 World Health Organization (WHO) classification.[23] Clinical information was obtained from patient medical charts. The use of clinical information and materials was approved by the Research Ethics Committee of Kurume University and was in accordance with the Declaration of Helsinki. According to the committee, informed consent was obtained.

In this study, all STS patients were provided with initial diagnoses, and underwent surgical complete resection with confirmed microscopic negative surgical margins. Cases with disease recurrence, synchronous metastasis, or who had received neoadjuvant therapies prior to surgical resection were excluded. All of the patients underwent periodical clinical follow-up at least every other year after resection (range, 0–146 months).

### Determination of GATA3 expression in soft tissue sarcomas

Each sarcoma sample was cut in maximum cross section. All of those samples were stained with hematoxylin and eosin for morphological investigation. We evaluated the pathomorphism of tumor cells by light microscope in slides made from those sections and selected the slide including most characteristic pathological features for the present study.

A GATA3 primary antibody (1:50, rabbit monoclonal, D13C9, Cell Signaling Technology, Danvers, MA) was used for IHC analysis. The detailed IHC protocol for GATA3 is as follows: FFPE tissue samples were sectioned at a thickness of 2.5 μm, and deparaffinized in xylene followed by 95% alcohol. After rehydration with H_2_O, antigen retrieval was performed with Tris-ethylenediaminetetraacetic acid buffer (pH 9.0) in a microwave oven at 95°C for 40 minutes. Endogenous peroxidase activity was blocked by incubation in 3% H_2_O_2_ solution for 5 minutes, followed by incubation with the GATA3 primary antibody for 60 minutes at room temperature. Samples were then incubated with an EnVision Detection Systems (Dako) secondary antibody for 30 minutes. Visualization of GATA3 was performed using diaminobenzidine (DAB) for 4 minutes.

The authors analyzed STS samples to define GATA3 positivity in each case based on the propensity of GATA3-positive neoplastic cells within all neoplastic cells in a ≥ 5-mm^2^ area. A neoplastic cell was defined as positive when the nucleus was stained at least weakly Authors detected the intensity of those staining by using criteria of other malignancy.[24] In all cases, the characterization of GATA3 positivity was evaluated using an optical microscope under 400-fold magnification in the field that showed the strongest immunoreaction of GATA3 in tumor regions. Vascular endothelial cells were used as negative control and Th2 type T-cells were done as positive control in immunohistochemistry of GATA3. Two independent observers (OK and MH) assessed GATA3 expression without any previous knowledge of clinical information.

The GATA3 positivity cutoff value was determined to be 4%, which was the median value of all STS cases in this study. Cases with a value > 4% were defined as GATA3-positive cases, while those with a value ≤ 4% were defined as GATA3-negative cases.

### Immunohistochemical detection of cyclin D1 and ErbB2 /HER2 expression

Primary antibodies used for immunohistochemistry were as follows: rabbit monoclonal anti-Cyclin D1 (1:100, M3642, DAKO, Tokyo, Japan): mouse monoclonal anti-ErbB2/HER2 (1:400, 29D8, Cell Signaling Technology, Danvers, MA). They were used for IHC analysis. The cutoff value of cyclin D1 and ErbB2/HER2 positivity was determined to be 10%.[25] Cases with the value > 10% were defined as positive cases, while those with the value ≤ 10% were done as negative cases.

### Statistical methods

Clinicopathological characteristics for the statistical comparison included prognostic factors identified in previous reports, including sex, age (< 60 or ≥ 60 years),[26] tumor size (≤ 5 cm or > 5 cm),[26–28] tumor depth (superficial or deep),[26] and FNCLCC histological grade (grade 1 or 2/3), tumor differentiation (score 1 or 2/3), mitotic count (0-9/10 or ≥ 10/10 high-power fields [HPF]), and degree of tumor necrosis (< 50% or ≥ 50%).[4,28]

The statistical association between clinicopathological characteristics and GATA3 expression was analyzed by chi-square test or Fisher’s exact test (two-tailed test). Disease-free survival (DFS) and OS were defined as the intervals between the day of pathological diagnosis and recurrence or death, respectively. DFS and OS curves were calculated using the Kaplan-Meier method, and a log-rank test was applied to evaluate statistical differences. Univariate and multivariate analyses were performed by a Cox proportional hazards model to assess the influence of each variable on DFS and OS. A *P*-value < 0.05 was considered to indicate statistical significance. Statistical analyses performed in this study were conducted using JMP software, version 11 (SAS institute, Tokyo, Japan).

## Result

### Clinicopathological characteristics

Histological types according to the WHO histological classification and clinicopathological features of all cases included in this study are shown in Tables 1 and 2, respectively. This study included 45 males (59.2%) and 31 females (40.8%) with a median age of 58.9 years (range, 8–88 years). The mean tumor size was 8.55 cm (range, 1–25 cm). With tumor orientation demarcated by the muscular fascia, superficial tumors were observed in 25 cases (32.9%), while deep tumors were observed in 51 cases (67.1%). FNCLCC histological grade was evaluated as score 1 in 12 cases (15.8%), score 2 in 24 cases (31.6%), and score 3 in 40 cases (52.6%). Positive expression of cyclin D1 and ErbB2/HER2 were seen in 44 cases (57.9%) and 0 case (0%) of sarcomas, respectively. Regarding additional treatment after resection, no additional therapy was performed in 49 cases (64.5%), radiotherapy was given in 14 cases (18.4%), chemotherapy was given in 8 cases (10.5%), and chemoradiotherapy was given in 5 cases (6.6%). During the follow-up period (mean, 51.1 months; range, 1–146 months), 16 cases (21.1%) died of local recurrence or distant metastasis.

**Table 1:** Patient Characteristics.

Characteristic		All (n = 76) (%)
Sex	Male	45 (59.2%)
	Female	31 (40.8%)
Age (years old)	Mean [range]	58.9 [8–88]
	≤ 60	33 (43.4%)
	> 60	43 (56.6%)
Primary tumor size (cm)	Mean [range]	8.55 [1–25]
	≤ 5	19 (25.0%)
	> 5	57 (75.0%)
Depth	Superficial	25 (32.9%)
	Deep	51 (67.1%)
FNCLCC histological grade	1	12 (15.8%)
	2	24 (31.6%)
	3	40 (53.6%)
Tumor differentiation	1	7 (9.2%)
	2	26 (34.2%)
	3	43 (56.6%)
Mitotic count	0-9/10HPF	29 (38.2%)
	10-19/10HPF	15 (19.7%)
	> 19/10HPF	32 (42.1%)
Tumor necrosis	No necrosis	21 (27.6%)
	< 50%	51 (67.1%)
	≥ 50%	4 (5.3%)
Cyclin D1	Negative	32(42.1%)
	Positive	44(57.9%)
ErbB2/HER2	Negative	76(100%)
	Positive	0(0%)
Initial treatment	Surgery alone	49 (64.5%)
	Surgery followed by radiotherapy	14 (18.4%)
	Surgery followed by chemotherapy	8 (10.5%)
	Surgery followed by chemoradiotherapy	5 (6.6%)
Follow up (months)	Mean [range]	51.1 [1–146]
Prognosis	Tumor death	16 (21.1%)
	Non-tumor death	2 (2.6%)
	Alive	58 (76.3%)

HPF, high-power fields; FNCLCC, French Fédération Nationale des Centres de Lutte Contre le Cancer.

**Table 2:** Histological type of STS.

Histological type	All (n = 76) (%)
Undifferentiated/unclassified sarcomas	19 (24.5%)
Myxoid liposarcoma	11 (14.5%)
Well differentiated liposarcoma	7 (9.2%)
Dedifferentiated liposarcoma	2 (2.6%)
Leiomyosarcoma	10 (13.2%)
Myxofibrosarcoma	10 (13.2%)
Synovial sarcoma	3 (3.9%)
Malignant peripheral nerve sheath tumor	4 (5.3%)
Extraskeletal osteosarcoma	3 (3.9%)
Fibrosarcoma	3 (3.9%)
Alveolar soft part sarcoma	2 (2.6%)
Epithelioid sarcoma	1 (1.3%)
Rhabdomyosarcoma	1 (1.3%)

STS, soft tissue sarcomas.

### Immunohistochemical detection of GATA3 expression in soft tissue sarcoma

**Fig 1:**
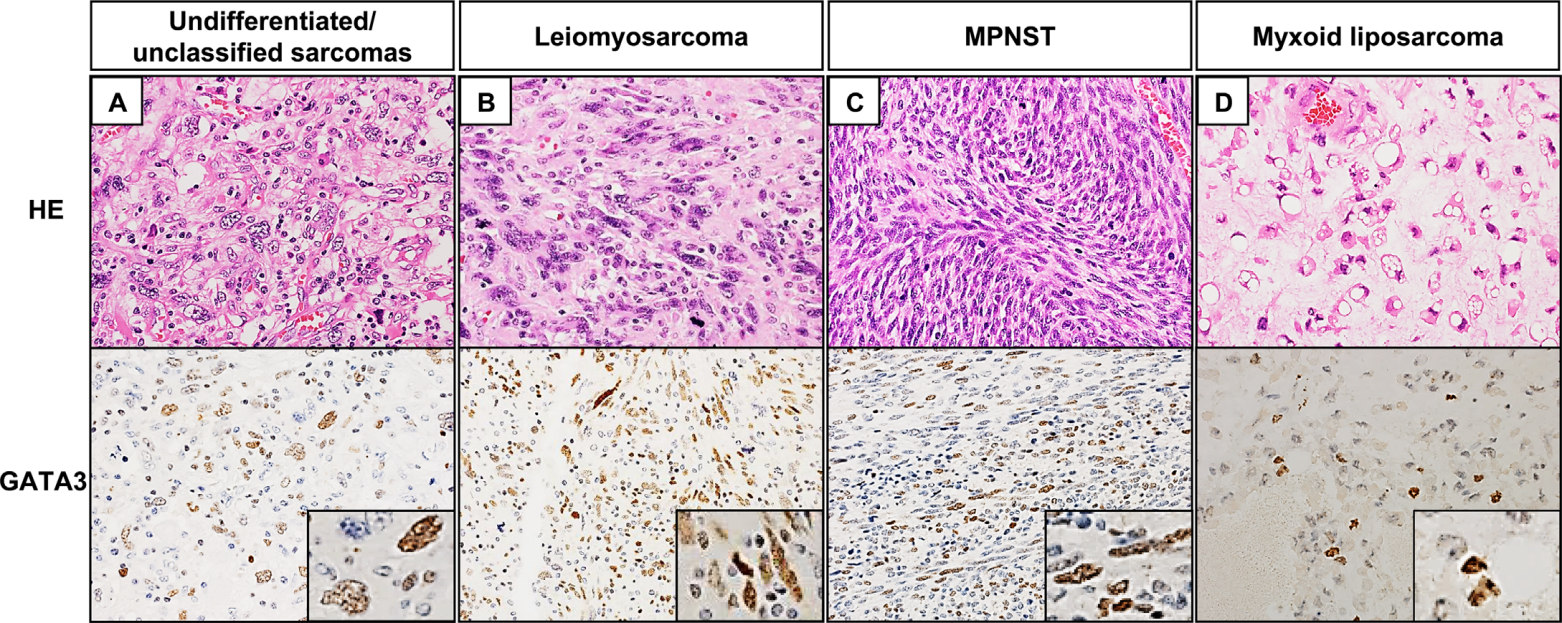
Immunohistochemical detection of GATA3 expression in soft tissue sarcomas (Original magnification, x400; insert, x600). GATA3 was present in the nuclei of STS cells at various proportions. (A) An undifferentiated/unclassified sarcomas case with 30% GATA3 expression. (B) A leiomyosarcoma case with 80% GATA3 expression. (C) A malignant peripheral nerve sheath tumor case with 70% GATA3 expression. (D) A myxoid liposarcoma case with 7% GATA3 expression.

**Fig 2:**
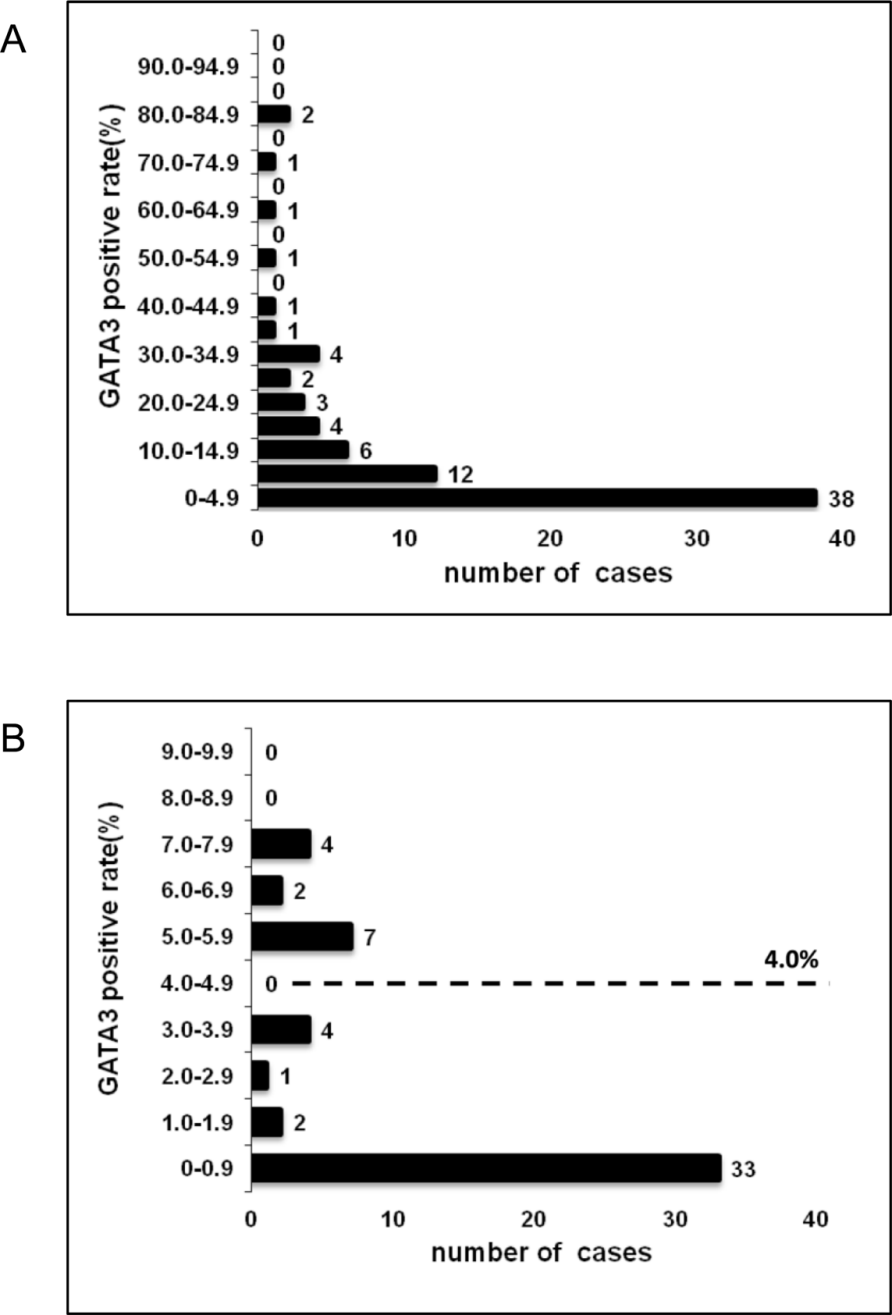
The distribution of GATA3 positive rate in soft tissue sarcomas. (A) The histogram of the GATA3 positive rate shows skewed distribution in all cases. (B) The histogram of GATA3 which focuses the percentage from 0 to 10 with the longitudinal axis by every 1 percent.

Representative tissue samples are shown in Fig 1. GATA3 expression was detected only within nuclei of neoplastic cells in all cases. Fig 2 showed the distribution of the GATA3 positive rate. The median GATA3 positivity value was 4% (range, 0–80%). Any tendencies between GATA3 expression and morphological and immunohistochemical findings was not observed. With respect to histological type, GATA3-positive expression was observed in 47.3% (9/19) of undifferentiated/unclassified sarcomas cases, 18.1% (2/11) of myxoid liposarcoma cases, 42.9% (3/7) of well differentiated liposarcoma cases, 50.0% (1/2) of dedifferentiated liposarcoma cases, 80.0% (8/10) of leiomyosarcoma cases, 40.0% (4/10) of myxofibrosarcoma cases, 100.0% (3/3) of synovial sarcoma cases, 75.0% (3/4) of malignant peripheral nerve sheath tumor cases, 66.7% (2/3) of extraskeletal osteosarcoma cases, 66.7% (2/3) of fibrosarcoma cases, 0.0% (0/2) of alveolar soft part sarcoma cases, 100.0% (1/1) of epithelioid sarcoma cases, and 0.0% (0/1) of rhabdomyosarcoma cases.

### Statistical association between GATA3 expression and clinicopathological characteristics in soft tissue sarcoma

Table 3 shows the statistical association between clinicopathological characteristics and GATA3 expression in this study. GATA3-positive expression significantly associated with a higher number of mitotic counts (*P* < 0.0001). In contrast, no significant differences in sex (*P* = 0.815), age (*P* = 0.247), tumor size (*P* = 0.791), depth (*P* = 0.807), FNCLCC histological grade (*P* = 0.113), tumor differentiation (*P* = 1.000), degree of tumor necrosis (*P* = 0.615), cyclinD1-positive (P = 0.1623), or ErbB2/HER2-positive (P-value, not calculable).

**Table 3:** Statistical association between clinical characteristics and GATA3 expression.

Characteristic		GATA3 positive n = 38 (50.0%)	GATA3 negative n = 38 (50.0%)	*P*-value (Chi-square)
Sex	Male	22	23	0.815
	Female	16	15	
Age (years old)	≤ 60	14	19	0.247
	> 60	24	19	
Primary tumor size (cm)	≤ 5	9	10	0.791
	> 5	29	28	
Depth	Superficial	13	12	0.807
	Deep	25	26	
FNCLCC histological grade	1	3	9	0.113^※^
	2&3	35	29	
Tumor differentiation	1	3	4	1.000^※^
	2&3	35	34	
Mitotic count	0-9/10HPF	6	23	<0.0001
	≥ 10/10HPF	32	15	
Tumor necrosis	< 50%	37	35	0.615^※^
	≥ 50%	1	3	
cyclin D1	Negative	19	25	0.1623
	Positive	19	13	
ErbB2 /HER2	Negative	38	38	
	Positive	0	0	

^※^*p*-value derived from Fisher's exact test.

### Disease-free survival according to GATA3 expression in soft tissue sarcoma

**Fig 3:**
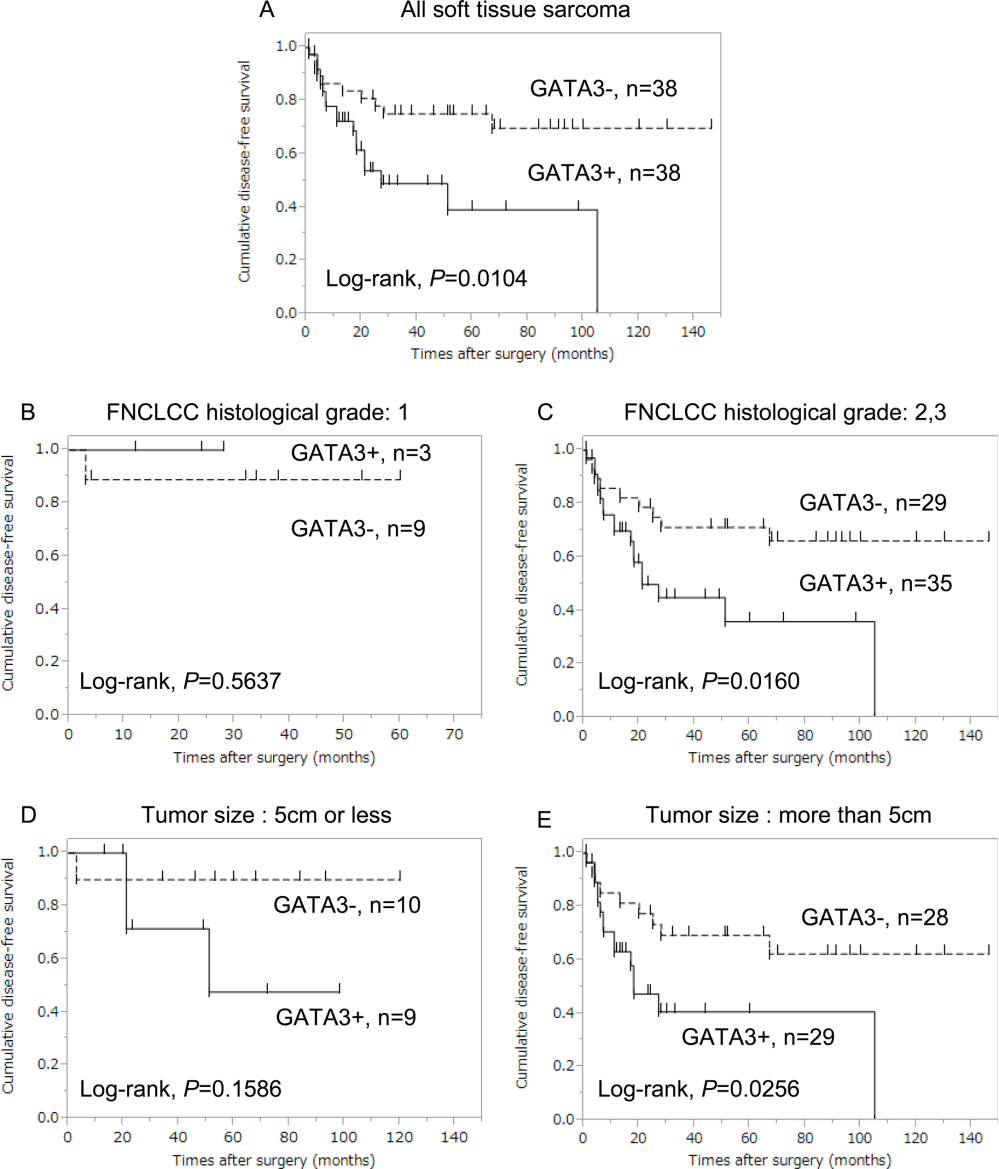
Comparison of disease-free survival curves between GATA3-positive (continuous line) and -negative (dotted line) STS cases. (A) Among all STS cases, GATA3-positive cases experienced significantly worse DFS compared to GATA3-negative cases (*P* = 0.0104). (B) Among FNCLCC histological grade 1 cases, no significant differences were observed between GATA-3 positive and -negative cases. (C) Among FNCLCC histological grade 2/3 cases, GATA3-positive cases experienced significantly worse DFS compared to GATA3-negative cases (*P* = 0.0160). (D) Among cases with tumors ≤ 5 cm, no significant differences were observed between GATA-3 positive and -negative cases. (E) Among cases with tumors > 5 cm, GATA3-positive cases experienced significantly worse DFS compared to GATA3-negative cases (*P* = 0.0256).

Fig 3 showed the DFS curves by GATA3 expression in STS cases. The DFS of GATA3-positive cases was significantly shorter than that of GATA3-negative cases (*P* = 0.0104) (Fig 3A). In the analysis stratified by FNCLCC histological grade, the DFS curves were not statistically different between GATA3-positive and -negative cases among FNCLCC histological grade 1 cases (*P* = 0.5637) (Fig 3B). However, in among FNCLCC histological grade 2 and 3 cases, GATA3-positive cases experienced significantly shorter DFS compared to GATA3-negative cases (*P* = 0.0160) (Fig 3C). In the analysis stratified by tumor size, the DFS curves were not statistically different between GATA3-positive and -negative cases among cases with ≤ 5-cm tumors (*P* = 0.1586) (Fig 3D). However, among cases with > 5-cm tumors, GATA3-positive cases experienced significantly shorter DFS compared to GATA3-negative cases (*P* = 0.0256) (Fig 3E).

### Overall survival according to GATA3 expression in soft tissue sarcoma

**Fig 4:**
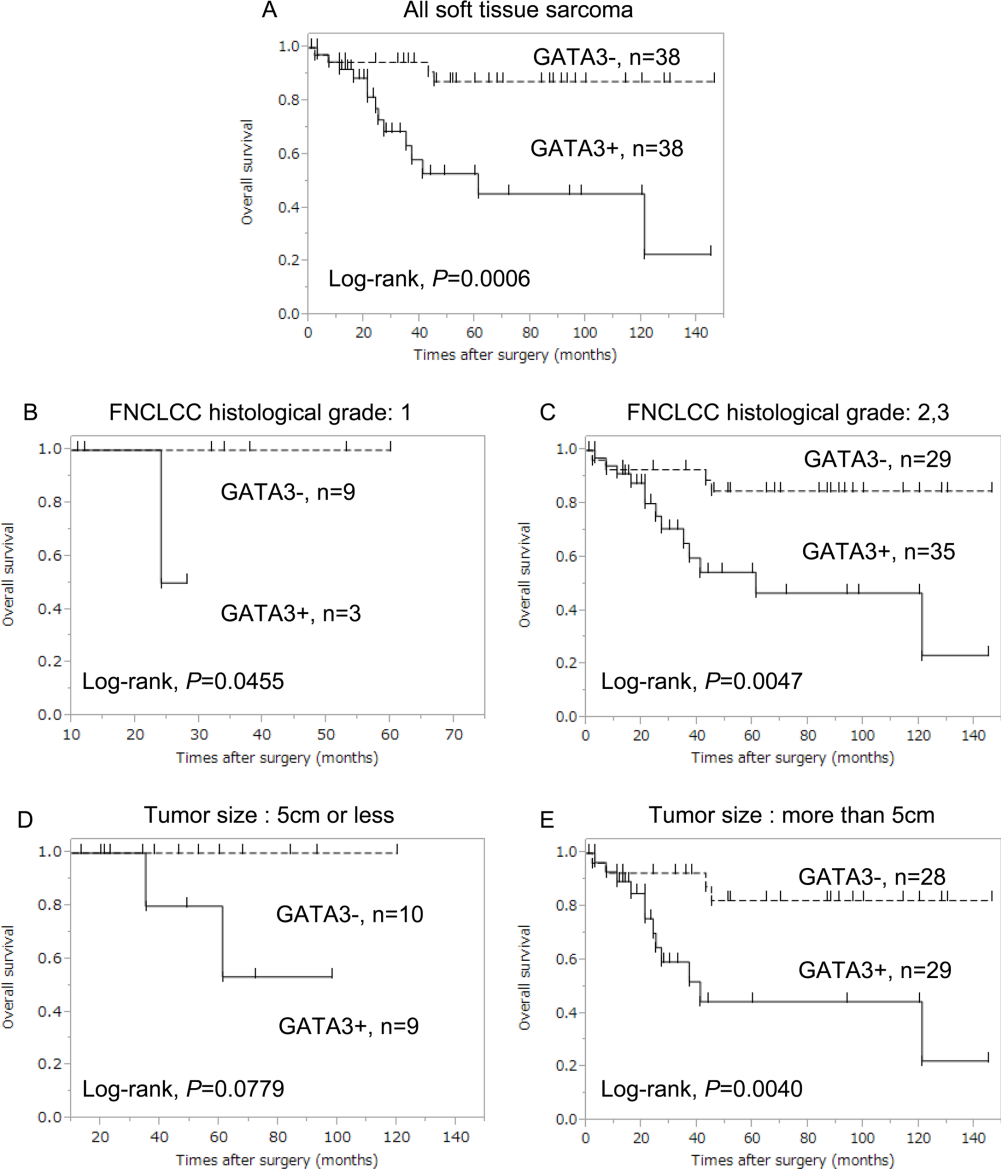
Comparison of overall survival curves between GATA3-positive (continuous line) and -negative (dotted line) cases. (A) Among all STS cases, OS for GATA3-positive cases was significantly worse than that for GATA3-negative cases (*P* = 0.0006). (B) Among FNCLCC histological grade 1 and (C) FNCLCC histological grade 2/3 cases, GATA3-positive cases experienced significantly worse OS compared to GATA3-negative cases (*P* = 0.0455 and *P* = 0.0047, respectively). (D) Among cases with tumors ≤ 5 cm, no significant differences were observed between GATA-3 positive and -negative cases. (E) Among cases with tumors > 5 cm, GATA3-positive cases experienced significantly worse OS compared to GATA3-negative cases (*P* = 0.0040).

OS curves by GATA3 expression are shown in Fig 4. OS for GATA3-positive cases was significantly shorter than that for GATA3-negative cases (*P* = 0.0006) (Fig 4A). In the analysis stratified by FNCLCC histological grade, GATA3-positive cases experienced significantly shorter OS compared to GATA-negative cases among both FNCLCC histological grade 1 cases (*P* = 0.0455) (Fig 4B) and grade 2/3 cases (*P* = 0.0047) (Fig 4C). When stratified by tumor size, the OS curves did not significantly differ between GATA3-positive and -negative cases among cases with tumors ≤ 5 cm (*P* = 0.0779) (Fig 4D). However, among cases with tumors > 5 cm, GATA3-positive cases experienced significantly shorter DFS compared to GATA3-negative cases (*P* = 0.0040) (Fig 4E).

### Univariate and multivariate analyses of disease-free survival and overall survival in relation to GATA3 expression in soft tissue sarcoma

The results of univariate and multivariate analyses for DFS and OS with respect to GATA3 expression are presented in Tables 4 and 5. Based on the DFS analysis, GATA3-positive expression was significantly associated with worse DFS in univariate analysis (hazard ratio [HR], 2.719; *P* = 0.012). GATA3-positive expression was also identified as an independent risk factor for recurrence in multivariate analysis (HR, 2.711; *P* = 0.014). For OS, GATA3-positive expression was significantly associated with worse OS in univariate analysis (HR, 5.730; *P* = 0.0007), and GATA3-positive expression was also identified as an independent poor prognostic factor for OS in multivariate analysis (HR, 5.789; *P* = 0.0008).

**Table 4:** Univariate and multivariate Cox regression analysis for disease-free survival in STS patients.

Characteristics	Univariate analysis			Multivariate analysis		
	Hazard ratio	95% confidence interval	*P* value	Hazard ratio	95% confidence interval	*P* value
Age (years old) (≤ 60 vs ≻ 60)	1.359	0.639–3.014	0.429	1.315	0.581–3.069	0.513
Depth (superficial vs deep)	0.875	0.407–1.986	0.740	0.906	0.400–2.141	0.817
Primary tumor size (cm) (≤ 5 vs > 5)	2.631	1.013–8.973	0.047	2.885	1.095–9.928	0.031
FNCLCC histological grade (1 vs 2&3)	5.195	1.101–92.795	0.035	4.047	0.852–72.432	0.087
GATA3 (positive vs negative)	2.719	1.249–6.270	0.012	2.711	1.219–6.384	0.014

STS, soft tissue sarcomas; FNCLCC,French Fédération Nationale des Centres de Lutte Contre le Cancer.

**Table 5:** Univariate and multivariate Cox regression analysis for overall survival in STS patients.

Characteristics	Univariate analysis			Multivariate analysis		
	Hazard ratio	95% confidence interval	*P* value	Hazard ratio	95% confidence interval	*P* value
Age (years old) (≤ 60 vs ≻ 60)	1.521	0.597–4.152	0.382	1.556	0.594–4.344	0.370
Depth(superficial vs deep)	0.879	0.340–2.530	0.798	1.059	0.399–3.121	0.912
Primary tumor size (cm) (≤ 5 vs > 5)	3.176	0.894–20.168	0.077	3.416	0.952–21.813	0.061
FNCLCC histological grade (1 vs 2&3)	2.650	0.533–48.004	0.277	1.373	0.264–25.197	0.752
GATA3 (positive vs negative)	5.730	2.024–20.418	0.0007	5.789	2.012–20.880	0.0008

STS, soft tissue sarcomas; FNCLCC,French Fédération Nationale des Centres de Lutte Contre le Cancer.

## Discussion

The results of this study show for the first time that GATA3-positive STS has significantly more mitotic counts compared to GATA3-negative STS. DFS and OS of GATA3-positive cases were significantly shorter than those of GATA-negative cases. Stratified analyses also indicated that GATA3-positive STS was associated with significantly shorter DFS and OS among cases with larger tumors and higher FNCLCC histological grades. Moreover, GATA3-positive expression was shown to be both an independent risk factor for recurrence and a poor prognostic factor for OS in multivariate analysis. The HR of GATA3-positive expression was highest among reported prognostic factors.[4,26–29] It cannot be denied that the relatively small number of cases in this study may have influenced these results. Nevertheless, GATA3 expression appears to be a better prognostic factor than previously identified factors.

As mentioned above, GATA3-positive cases experienced significantly shorter DFS compared to GATA3-negative cases. In multivariate analysis, GATA3-positive expression was also detected as an independent risk factor influencing recurrence, in addition to tumor size. Several studies have reported that tumor size[26,30,31] and FNCLCC histological grade[30] are independent predictors for distant metastasis in STS. In addition, Stojadinovic et al. reported that tumor size was also a significant risk factor for local recurrence.[32] The present results suggest that GATA3 expression may be valuable in the stratification of patients for predicting recurrence, including both local recurrence and distant metastasis after complete resection, particularly among cases with large tumors and/or a high FNCLCC histological grade.

Previous reports in other malignancies showed GATA3 expression was associated with activation of cyclin D1 and expression of ErbB2 /HER2, which might be leading to a poor prognosis. [17,20,21,33] However, there were no significant associations between GATA3 expression, and cyclinD1 or ErbB2/HER2. Although underlying mechanisms are not clear in STS, these results could emphasize the importance of GATA3 expression as a poor prognostic factor, because GATA 3 expression was independent from cyclinD1 and HER2.

Even recently, there is no general consensus about the evaluation method of the positivity cutoff value especially in GATA3. A definition of biologic cutoff value was also considered to be difficult. In the previous studies of breast cancer which set cutoff value according to IHC positivity, there is no consistency in determining GATA3 positivity among the studies. Each studies set cutoff value of GATA3 positivity to 20% [34], 10% [35] and 5% [36]. The reason why the cutoff value was adopted was not well declared in those manuscripts. In gastric adenocarcinoma, Keshari et al. defined that GATA3 immunostaining score was calculated as the sum of the percentage of positively stained tumor cells and the staining intensity. The score ranged from 0 to 9 and the cutoff value was set to be score 3.[14] There are several articles that define the median as cutoff value, although they were not about GATA3 expression.[37–39] Indeed, the histogram of GATA3 positivity in STS demonstrated skewed distribution as below (Fig 2A). Therefore, cutoff value in this study, which is the statistical median value, is considered to be appropriate.

This study has some limitations. First, the relatively small number of patients made it difficult to evaluate the association between GATA3 expression and clinicopathological features of each histological type. Additional studies including larger numbers of patients with each histological type are required; however, it should be noted that the majority of previous studies grouped STS patients using the FNCLCC histological grading system.[23] Second, GATA3 expression was investigated only by IHC analysis. Additional analyses of genomic abnormalities and/or transcriptional mechanisms are required to fully understand how GATA3 is expressed biologically.

In this study, GATA3 expression was shown to be an independent risk factor for recurrence after complete resection, as well as a prognostic factor for OS in STS patients. These results suggest that evaluation of GATA3 expression might contribute to more effective clinical strategies. Further studies are required to clarify the functions and mechanisms of GATA3 in STS.

## Supporting Information

S1 Table Data analyzed in this study. (XLSX)
